# Comparative transcriptome analysis of *Poncirus trifoliata* identifies a core set of genes involved in arbuscular mycorrhizal symbiosis

**DOI:** 10.1093/jxb/ery283

**Published:** 2018-07-26

**Authors:** Jianyong An, Mengqian Sun, Robin van Velzen, Chuanya Ji, Zijun Zheng, Erik Limpens, Ton Bisseling, Xiuxin Deng, Shunyuan Xiao, Zhiyong Pan

**Affiliations:** 1 Key Laboratory of Horticultural Plant Biology (Ministry of Education), Key Laboratory of Horticultural Crop Biology and Genetic Improvement (Central Region, Ministry of Agriculture), College of Horticulture and Forestry Sciences, Huazhong Agricultural University, Wuhan, P.R. China; 2 Department of Plant Sciences, Laboratory of Molecular Biology, Wageningen University, Droevendaalsesteeg, PB Wageningen, Netherlands; 3 Institute for Bioscience and Biotechnology Research & Department of Plant Sciences and Landscape Architecture, University of Maryland College Park, Rockville, MD, USA

**Keywords:** Arbuscular mycorrhiza, citrus, comparative transcriptome, *FatG*, *Glomus versiforme*, *Poncirus trifoliata*, RNA-seq

## Abstract

The perennial woody plants of citrus are one of the most important fruit crops in the world and largely depends on arbuscular mycorrhizal symbiosis (AMS) to obtain essential nutrients from soil. However, the molecular aspects of AMS in citrus and perennial woody plants in general have largely been understudied. We used RNA-sequencing to identify differentially expressed genes in roots of *Poncirus trifoliata* upon mycorrhization by the AM fungus *Glomus versiforme* and evaluated their conservation by comparative transcriptome analyses with four herbaceous model plants. We identified 282 differentially expressed genes in *P. trifoliata*, including orthologs of 21 genes with characterized roles in AMS and 83 genes that are considered to be conserved in AM-host plants. Comparative transcriptome analysis revealed a ‘core set’ of 156 genes from *P. trifoliata* whose orthologous genes from at least three of the five species also exhibited similar transcriptional changes during AMS. Functional analysis of one of these conserved AM-induced genes, a 3-keto-acyl-ACP reductase (FatG) involved in fatty acid biosynthesis, confirmed its involvement in AMS in *Medicago truncatula*. Our results identify a core transcriptional program for AMS that is largely conserved between *P. trifoliata* and other plants. The comparative transcriptomics approach adds to previous phylogenomics studies to identify conserved genes required for AMS.

## Teaser

A comparative transcriptome analysis of *Poncirus trifoliata* and four herbaceous model plants identifies a core set of arbuscular mycorrhiza-induced genes that are likely to play key roles in symbiosis.

## Introduction

Arbuscular mycorrhiza (AM) is a mutualistic symbiosis widely formed between AM fungi (AMF) and the roots of terrestrial plants (Lanfranco *et al.*, 2016). The fungal partner forms arbuscules in root cortical cells and facilitates uptake of mineral nutrients from the soil by the host plants (Smith and Smith, 2011; Wang *et al.*, 2017). AMF colonization can also improve plant tolerance to biotic (Pozo and Azcón-Aguilar, 2007; Song *et al.*, 2015) and abiotic stresses (Ruiz-Lozano *et al.*, 2016; Santander *et al.*, 2017). In return, the host plant provides sugars and lipids to the fungal partner (Rich *et al.*, 2017*b*; Roth and Paszkowski., 2017).

The perennial woody plants of citrus are one of the most important fruit crops in the world. Because citrus and its close relatives rarely have root hairs (Cao *et al.*, 2013), it is believed that uptake of mineral nutrients from the soil by the roots is largely dependent on symbiotic AMF (Davies and Albrigo, 1994; Wu *et al.*, 2016). Indeed, it has been shown that AMF can significantly promote growth performance of citrus trees (Chen *et al.*, 2014; Xiao *et al.*, 2014) and improve their tolerance to abiotic stresses (Wu and Xia, 2006). However, there has been virtually no mechanistic study of AM symbiosis (AMS) in citrus crops.

The establishment of AMS entails a number of molecular and cell developmental processes that are for a large part controlled by a signaling pathway that is highly conserved in AM host plants (Delaux *et al.*, 2013; Gutjahr and Parniske, 2013; Bravo *et al.*, 2016). This pathway is thought to be activated upon perception of fungal (lipo-)chitooligosaccharidic signal molecules and leads to the activation of AM-responsive genes (Maillet *et al.*, 2011; Czaja *et al.*, 2012; Genre *et al.*, 2013). Several key transcription factors have been identified that are essential to establish functional AMS (Pimprikar *et al.*, 2016). One of the key transcription factors that is essential for proper arbuscule development and induction of many of the AM-responsive genes is the evolutionarily conserved GRAS protein RAM1, which, for example, induces the expression of symbiotic phosphate transporter genes and multiple genes constituting a lipid biosynthesis pathway (Park *et al.*, 2015; Luginbuehl *et al.*, 2017; Rich *et al.*, 2017*a*).

A lot of studies aimed at identifying AM-induced genes have been performed in different plants. Studies in *Medicago truncatula*, *Lotus japonicus*, *Oryza sativa* (rice), *Solanum lycopersicum* (tomato), *Casuarina glauca*, *Petunia hybrida*, and *Vitis vinifera* have led to the identification of thousands of genes responsive to AMF colonization, of which many have shown specific expression in cortical cells containing arbuscules (Liu *et al.*, 2003; Wulf *et al.*, 2003; Grunwald *et al.*, 2004; Frenzel *et al.*, 2005; Deguchi *et al.*, 2007; Gomez *et al.*, 2009; Breuillin *et al.*, 2010; Hogekamp *et al.*, 2011; Gaude *et al.*, 2012; Tromas *et al.*, 2012; Hogekamp and Küster, 2013; Gutjahr *et al.*, 2015; Handa *et al.*, 2015; Balestrini *et al.*, 2017; Garcia *et al.*, 2017; Sugimura and Saito, 2017; Rich *et al.*, 2017*a*). However, the transcriptional reprogramming of citrus involved in symbiosis with AMF remains to be determined.

To gain insights into the genes involved in a successful AMS in citrus, we used RNA-sequencing technology to investigate AMF colonization-induced transcriptomic changes in roots of *Poncirus trifoliata* (synonym *Citrus trifoliata*), a close relative and the most common rootstock of cultivated citrus. We then used a comparative transcriptomics approach to assess the level of conservation in AMF-induced transcriptional reprogramming between *P. trifoliata* and four herbaceous model plants. This approach allowed us to identify a core set of genes that are co-regulated in diverse plant species, which included orthologs of most genes known to have characterized roles in AMS and the vast majority of genes that are conserved in AM-host plants. This comparative transcriptome analysis adds to previous phylogenomics approaches (Delaux *et al.*, 2014; Bravo *et al.*, 2016) aimed at identifying conserved genes required for AMS. Reverse-genetic analyses on one of the core genes, a 3-keto-acyl-ACP reductase (FatG), confirmed its essential role in AM symbiosis in *M. truncatula*.

## Materials and methods

### Plant growth, and mycorrhizal inoculation and visualization

Seeds of *Poncirus trifoliata* (L.) Raf (syn. *Citrus trifoliata*) were surface-sterilized by washing in 1 M NaOH for 15 min and in 2% sodium hypochlorite for 20 min, followed by rinsing with distilled water. The seeds were placed in Petri dishes and covered with sterilized gauze for 1 week in the dark (28 °C), and then sown in autoclaved vermiculite and placed in a greenhouse for 1 month. The seedlings were transplanted into sterile quartz sand with eight plants per pot (18 × 18 cm) and grown under greenhouse conditions (30/22 °C day/night). For mycorrhizal inoculation, the AM fungi *Glomus versiforme* was used because it was previously reported to show better colonization levels in *P. trifoliata* roots compared to other AM fungi (Shu *et al.*, 2012). The *G. versiforme* strain (BGC NM03C, Institute of Plant Nutrition and Resources, Beijing Academy of Agriculture and Forestry Sciences) was propagated on *Allium schoenoprasum* in sterile quartz sand. The sand containing spores, mycorrhizal roots, and extraradical mycelia was used as inoculum for *P. trifoliata* inoculation. A sample of 50 g sand containing about 3000 *G. versiforme* spores was added to each pot of *P. trifoliata* according to published protocols (Krajinski *et al.*, 2014; Huisman *et al.*, 2016). Plants without AM inoculum were used as controls. Plants were watered twice a week with 250 ml half-strength Hoagland solution containing 20 μM phosphorus as described previously (Shu *et al.*, 2012). Roots were collected every 2 weeks to assess the mycorrhizal colonization rates by staining with Trypan Blue (Trouvelot *et al.*, 1986) or wheat germ agglutinin (WGA) Alexa Fluor 488 (Ivashuta *et al.*, 2005). After 3 months post-inoculation, the *P. trifoliata* roots were well colonized and arbuscules were observed (Supplementary Fig. S1 at *JXB* online), and therefore plants were sampled at this time. Lateral roots from mycorrhizal and non-mycorrhizal plants were harvested for RNA-sequencing (RNA-seq) with three biological replicates, each containing at least nine plants collected from different pots.

For *Medicago truncatula*, hairy root-transformed plants were constructed (see below). Transformed plants were transferred to pots containing a mixture of sterile clay and sand (1:1 by volume) and watered twice a week with modified Hoagland medium containing 20 μM phosphorus (Javot *et al.*, 2011). For promoter analysis using GUS (β-glucuronidase) and RNAi assays, the plants were inoculated with commercial AM inoculum containing ~300 spores of *Rhizophagus irregularis* (DAO197198 strain, Agronutrition, France) per plant for 6 weeks. The inoculum only contained spores. Non-inoculated plants were used as controls.

### RNA-seq and data processing

Total RNA was extracted using TRIzol^®^ reagent (Invitrogen, USA) according to the manufacturer’s instructions. A TURBO DNAase reagent kit (Ambion, USA) was used to remove DNA contamination. The quality, quantity, and RNA integrity (RIN) number were measured using a Nanodrop ND 1000 spectrophotometer and an Agilent Technologies 2100 Bioanalyzer. Total RNA from six samples (three biological replicates of mycorrhizal and non-mycorrhizal samples) were used for RNA-seq with an Illumina HiSeq^TM^ 2000 at the Beijing Genomics Institute (Shenzhen). First, total RNA from each sample was used to enrich mRNA using oligo (dT) magnetic beads, and subsequently used for the synthesis of double-strand cDNA (Mortazavi *et al.*, 2008). The double-strand cDNA was purified, washed in EB buffer for end repair, added with single-nucleotide A (Adenine), and ligated with sequencing adaptors. The fragments obtained were used for PCR amplification, followed by library construction and sequencing. An Agilent 2100 Bioanalyzer and the ABI StepOnePlus Real-Time PCR System were used for checking the quality and quantity of the library. After sequencing, low-quality reads (>50% of the bases with a quality value Q≤5) or reads containing more than 10% of unknown bases were filtered. The resulting clean reads were then aligned to the *Citrus sinensis* reference genome (Xu *et al.*, 2013) (http://citrus.hzau.edu.cn/orange) using the software SOAPaligner/SOAP2 (Li *et al.*, 2009) allowing two bp mismatches per read. We note that this approach excluded genes that are not present in the *C. sinensis* genome, or which are divergent between *C. sinensis* and *P. trifoliata* /*C. trifoliata*; nevertheless, approximately 74% of the RNA-seq reads could be mapped, which was similar to previous RNA-seq analyses for *Poncirus* (Terol *et al.*, 2016; Chen *et al.*, 2017; Supplementary Table S1). Gene expression levels were calculated using the RPKM (reads per kb per million reads) method as follows: RPKM = 10^6^ × [(Number of reads uniquely aligned to gene A)/(Total number of reads that uniquely aligned to all genes)] × [(Number of bases of gene A)/10^3^] (Mortazavi *et al.*, 2008). The differentially expressed genes (DEGs) and their probability (*P*) were calculated by using the NOIseq method (Tarazona *et al.*, 2011). Only the genes whose expression levels change ≥2-fold and *P*≥0.8 were defined as DEGs, as previously reported (Tarazona *et al.*, 2011). The threshold *P*≥0.8 is equivalent to the odds of (probability of a gene being a DEG)/(probability of a gene being a non-DEG) being >4.

### Orthogroup inference and comparative expression analysis

To determine the relationships between genes from five AM host species, as well as with other plant species, we inferred orthogroups using OrthoFinder (Emms and Kelly, 2015). The algorithm used by the OrthoFinder software reduces gene length bias and outperforms the most widely used method, OrthoMCL (Li *et al.*, 2003) in accuracy and speed. Since orthogroups are defined as the set of genes that are descended from a single gene in the last common ancestor of all the species being considered, they can comprise orthologous as well as paralogous genes. Our analysis included proteomes from the selected AM host plants *C. sinensis*, *M. truncatula*, *L. japonicus*, *S. lycopersicum*, and *O. sativa*, as well as from the non-host plant *Arabidopsis thaliana* and the basal angiosperm *Amborella trichocarpa*. Consequently, each orthogroup comprised all descendants of a single gene in the most recent common ancestor of Angiosperms.

To assess common utilization of genes in the five AM host species, we compared the set of genes that are differentially expressed between AMF-colonized roots and uninoculated controls from *Poncirus*/Citrus with similar sets published for *Medicago*, *L. japonicus*, *O. sativa*, and *S. lycopersicum* (Fiorilli *et al.*, 2009; Hogekamp *et al.*, 2011; Gaude *et al.*, 2012; Gutjahr *et al.*, 2015; Handa *et al.*, 2015; Garcia *et al.*, 2017; Sugimura and Saito., 2017). Sets were compared based on gene orthogroup membership such that any orthogroup comprising DEGs from different AM host species were scored as common utilization.

### Quantitative RT-PCR

First-strand cDNAs were synthesized from RNA using a RevertAid^TM^ First Strand cDNA Synthesis Kit (Thermo Scientific). The gene-specific primers (Supplementary Table S2) were designed using the Primer Express software (PE applied Biosystems, USA). Reactions containing 500 ng of cDNA with gene-specific primers and SYBR Green PCR Master Mix in a 10-μl reaction were performed in an ABI 7900HT Fast Real-time system. The thermal profiles were: 50 °C for 2 min, 95 °C for 1 min, then 95 °C for 15 s, and 60 °C for 1 min for 40 cycles, followed by generation of a melting-curve. The value of threshold cycle (*C*t) was used to calculate the transcript abundance relative to the housekeeping gene translation initiation factor 1alpha (*P. trifoliata* gene *eIF1alpha*). This reference gene showed more stability than others in *P. trifoliata* mycorrhizal and non-mycorrhizal samples and thus it was selected out from a series of commonly used citrus reference genes (Liu *et al.*, 2013; Wu *et al.*, 2014). For *M. truncatula* samples, the widely used housekeeping gene *MtEF1* was used as the reference.

### Plasmid construction and hairy root-transformed plants of *M.truncatula*

The promoter fragments of the selected *P. trifoliata* (*Ptr*) genes, i.e. *PtrChit2*, *PtrEXO70*, *PtrPMI2*, *PtrLipase3*, and *PtrFatG*, which correspond to 815, 1305, 1235, 1233, and 1915 bp upstream of the translation initiator ATG of the respective genes, were cloned into the pENTR-TOPO^TM^ vector or pDONR 221 vector (Invitrogen) by TOPO or BP reaction using specific primers (Supplementary Table S3). For promoter-GUS analysis, a binary vector pKGWFS2-RR (Op den Camp *et al.*, 2011) including the DsRed expression cassette as a visual marker was used. Subsequently, the promoters of *PtrChit2*, *PtrExo70*, *PtrPMI2*, *PtrLipase3*, and *PtrFatG* were shuffled to the pKGWFS2-RR vector by LR reaction using Gateway^®^ LR Clonase^TM^ II (Invitrogen).

For RNAi analysis, a ~429-bp coding sequence of *MtFatG* (Medtr4g097510) was cloned into the pENTR/D-TOPO^TM^ vector using gene-specific primers (Supplementary Table S3), followed by recombination into the pK7GWIWG2(II) binary vector containing DsRed as a visual selection marker (Limpens *et al.*, 2004) using Gateway^®^ LR Clonase^TM^ (Invitrogen). An empty construct CHEAP-pK7GWIWG2 (II) was used as a negative control in the hairy root transformation assays (Limpens *et al.*, 2004).

All the recombinant plasmids were confirmed by sequencing and introduced into *Agrobacterium rhizogenes* (recently re-classified as *Rhizobium rhizogenes*; Ormeño-Orrillo *et al.*, 2015) strain MSU440 by electroporation, and the positive *Agrobacterium* cells were used for hairy root transformation in *M. truncatula* A17 (Limpens *et al.*, 2004).

### WGA and GUS staining, microscopy, and quantification of AMF colonization

For RNAi analysis, the *M. truncatula* transformed roots were cleaned with water, incubated in 10% (w/v) KOH at 98 °C for 40 min, washed three times in PBS solution, and then incubated in PBS solution containing 0.2 μg ml^–1^ WGA Alexa Fluor 488 at 4 °C overnight. For imaging of arbuscules, confocal microscopy was used (Leica Confocal TCS-SP8, 40× water objective). Roots were cut into 1-cm fragments, and the level of colonization was calculated according to the previously reported method of Trouvelot *et al.* (1986). At least 30 of the 1-cm root fragments were randomly picked and assessed according the class of mycorrhizal colonization and the abundance of arbuscules. The colonization level was expressed by the following parameters: frequency of the mycorrhizae in the root system (F); intensity of the mycorrhizal colonization in the root fragments (m); intensity of the mycorrhizal colonization in the root system (M), arbuscule abundance in mycorrhizal parts of root fragments (a); and arbuscule abundance in the root system (A). All the parameters are expressed as percentages.

For promoter analysis, GUS and WGA Alexa Fluor 488 co-staining was conducted as follows. The transgenic roots were harvested 1 month after inoculation with *R. irregularis* stained with GUS solution at 37 °C for 3–5 h, and then washed three times in water. The roots were then boiled in 10% KOH and WGA 488 staining was conducted as indicated above. GUS and WGA 488 imaging were performed using a Nikon fluorescence microscope. Root segments already stained by GUS solution were embedded in Technovit 7100 (Xiao *et al.*, 2014), sectioned longitudinally (10 μm) using a microtome (RJ2035, Leica), stained in 0.1% Ruthenium Red for 15 min, and then imaged using a Lecia DM5500B microscope.

### Accession numbers

Sequence data from this study can be found in the GenBank database under the following accession numbers: promoter sequence of PtrExo70I (PtrExo70I_pro_, KU664538); PtrPMI2_pro_ (KU664542); PtrChit2_pro_ (KU664543); PtrLipase3_pro_ (KU664544); PtrFatG_pro_ (MH290725). RNA-seq data were deposited in the NCBI GEO (GSE77455).

## Results

### Transcriptomic analysis identified 282 *P. trifoliata* genes responsive to colonization by *Glomus versiforme*

In order to identify AM-responsive genes in the important citrus rootstock *P. trifoliata*, total RNA was extracted from lateral roots of plants colonized by *G. versiforme* (*Gv*) at 3 months post-inoculation (mpi), and from corresponding roots of non-inoculated control plants, and the samples were subjected to RNA-seq analysis. The frequency of mycorrhizae in the roots (F%) was ~80%, with an arbuscule abundance (A%) of 18%, and well-developed arbuscules were observed (Supplementary Fig. S1). Among the 28195 annotated genes in the citrus genome, a total of 22589 were detected in all the biological replicates (Supplementary Table S4).

We found that 245 genes were up-regulated and 37 were down-regulated in the AMF-colonized samples when compared to controls (Supplementary Table S5; ≥2-fold change, *P*≥0.8). Among the most *Gv*-induced *P. trifoliata* genes were two genes (Cs7g29450 and Cs9g18560) that are orthologous to *MtPT4*, which encodes a symbiotic phosphate transporter that is strongly induced by AMF and is essential for symbiotic phosphate uptake in the model plant *M. truncatula* (Javot *et al.*, 2007).

To validate our RNA-seq data, we selected 36 genes that showed elevated expression levels in *Gv*-colonized roots and examined their expression by quantitative RT-PCR using independent samples. The results showed that all of these genes did indeed display higher expression levels in *Gv*-colonized root samples compared to non-inoculated controls (Supplementary Fig. S4A, Supplementary Table S6), confirming the RNA-seq data.

### The *P. trifoliata* AMS-regulated gene set is highly enriched for known AMS-associated genes

To assess to which extent the *P. trifoliata* transcriptome data covered the genes known to be implicated in AMS, we searched the data for 24 genes reported to be functionally associated with, and up-regulated in, AMS (Supplementary Table S7), as well as for orthologs of the 138 *M. truncatula* genes that are reported to be conserved in AMF host plants but absent in non-host plants, as identified by a phylogenomics approach (Bravo *et al.*, 2016).

**Fig. 1.**
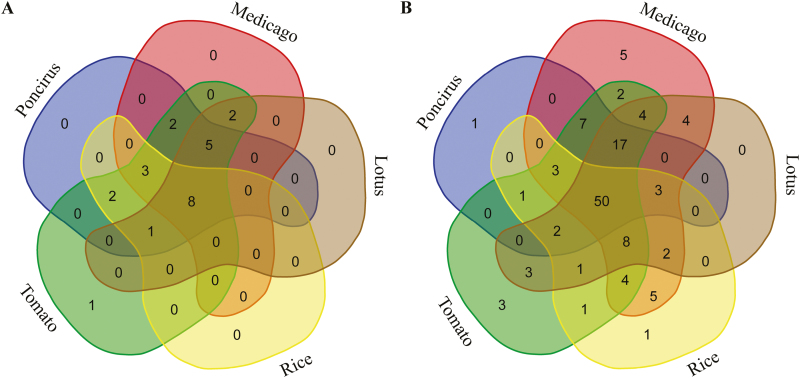
Distribution of orthologs of 24 known AMS genes and 138 AM-conserved *Medicago truncatula* genes in *Poncirus trifoliata*, *M. truncatula*, *Lotus japonicus*, *Oryza sativa*, and *Solanum lycopersicum*. (A) Venn diagram showing the number of known AMS genes up-regulated in the transcriptome data of the five AM host plants. (B) Venn diagram showing the number of 138 AMS conserved *M. truncatula* genes (Bravo *et al.*, 2016) containing orthologs that are similarly up-regulated in the transcriptome data of the five AM host plants. The transcriptome data in (A, B) are based on *M. truncatula* (Hogekamp *et al.*, 2011; Gaude *et al.*, 2012; Garcia *et al.*, 2017), *L. japonicus* (Handa *et al.*, 2015), *S. lycopersicum* (Fiorilli *et al.*, 2009; Sugimura and Saito., 2017), and *O. sativa* (Gutjahr *et al.*, 2015).

*Poncirus trifoliata* orthologs of 21 of the selected 24 AMS-associated genes that are up-regulated in herbaceous plants during AMS (*PT4*, *Exo70I*, *FatM*, *KIN3*, *RAM1*, *RAM2*, *STR*, *STR2*, *RAD1*, *VAPYRIN*, *HA1*, *KIN5*, *CYT733A1*, *KIN2*, *DISI/KASII*, *MYB1*, *OsNOPE*, *ERF1*, *PDR1*, *SbtM1*, *SbtM3*) were also found to be up-regulated in AMF-colonized *P. trifoliata* roots (Fig. 1A, Supplementary Tables S7, S12). Two AMS-associated genes that did not show up-regulation in our transcriptome analysis were *MIG1* (Heck *et al.*, 2016) and *RFCb* (Bravo *et al.*, 2016), while *Poncirus*/citrus lacks a true ortholog of the GRAS transcription factor DIP1(Yu *et al.*, 2014) (Supplementary Table S8).

Among the 138 *M. truncatula* genes that have orthologs conserved in AMF host plants (Bravo *et al.*, 2016), 102 had orthologs (based on orthogrouping, see below) that showed up-regulation in at least two additional AM-host plants (Fig. 1B). Notably, orthologs of 84 of these 138 genes also showed up-regulation in AMF-colonized roots of *P. trifoliata* (Fig. 1B). These results indicated that AMF-induced transcriptional reprogramming has a core set of genes conserved between *P. trifoliata* and the four model plants, which are likely to have key functions in AM symbiosis.

### Conservation of cis-regulatory elements in AMS-induced genes

Many AMF-induced genes are highly expressed in arbuscule-containing cells (Hogekamp *et al.*, 2011; Gaude *et al.*, 2012). To assess whether orthologous genes from *P. trifoliata* also showed a similar spatial expression pattern as their herbaceous counterparts, we studied whether the cis-regulatory elements in the corresponding *P. trifoliata* promoter regions resulted in similar arbuscule-enriched expression patterns. We performed promoter-*GUS* analyses by heterologous expression of five *P. trifoliata Gv*-induced genes in *M. truncatula*. These five genes are predicted to encode a subunit protein of the EXO70 family protein member (*PtrExo70I*, Cs3g16120), a type-2 chitinase (*PtrChit2*, Cs1g05710), a protein plastid movement impaired 2 isoform (*PtrPMI2*, Cs6g18300), a lipase class 3 (*PtrLipase3*, Cs2g28830), and a 3-ketocyl-ACP reductase (*PtrFatG*, Cs1g21320). We introduced each of the five *P. trifoliata* promoter-*GUS* constructs into roots of *M. truncatula* A17 by *Agrobacterium rhizogenes*-mediated transformation and assessed for GUS activity in transgenic roots inoculated with *R. irregularis* and non-inoculated control roots. All the selected *P. trifoliata* promoters resulted in arbuscule-enriched GUS expression (Supplementary Figs S2, S3), in line with the predicted expression pattern of the orthologous genes from *M. truncatula* (Gaude *et al.*, 2012; Hogekamp and Küster, 2013). By manually analysing the promoter sequences of the five *P. trifoliata* genes and their othologous genes in *M. truncatula*, we found all the five pairs of promoters contained one known mycorrhizal-related cis-regulatory motif MYCS/CTTC (Chen *et al.*, 2011; Lota *et al.*, 2013) or CTTC-like motifs with a single nucleotide mismatch (Supplementary Table S9). This indicated that the cis-regulatory elements required for arbuscule-enriched expression are largely conserved between *P. trifoliata* and *M. truncatula*.

Taken together, these results strengthen the notion that a core AMF-induced transcriptional program is evolutionary conserved.

### Comparative transcriptome analyses reveal conservation in transcriptional reprogramming during AMS

To further investigate the extent to which the transcriptional reprogramming upon AMS is conserved in the five flowering plant species (*P. trifoliata*, *M. truncatula*, *L. japonicus*, *S. lycopersicum*, and *O. sativa*), we performed a wider comparative transcriptome analysis. To determine orthologous relationships for AMF-induced DEGs, we first established a total of 15239 orthology groups (orthogroups) using OrthoFinder (Emms and Kelly, 2015) based on available proteomes (see Methods). The construction of orthogroups included proteomes from the five selected AM host plants as well as from *A. thaliana* and the basal angiosperm *Am. trichocarpa*. These orthogroups contained 23872 (81% of the whole genome gene) citrus genes, 41200 (71%) *M. truncatula* genes, 25235 (64%) *L. japonicus* genes, 21410 (51%) *S. lycopersicum* genes, and 25234 (51%) *O. sativa* genes. Next, we identified orthogroups containing AMF-induced genes from each of the five host plants and compared their expression patterns based on available data sets; representing in total 2368, 2124, 996, 1839 up-regulated genes and 324, 431, 446, 2612 down-regulated genes from *M. truncatula* (Hogekamp *et al.*, 2011; Gaude *et al.*, 2012; Garcia *et al.*, 2017), *L. japonicus* (Handa *et al.*, 2015), *S. lycopersicum* (Fiorilli *et al.*, 2009; Sugimura and Saito, 2017), and *O. sativa* (Gutjahr *et al.*, 2015), respectively.

**Fig. 2.**
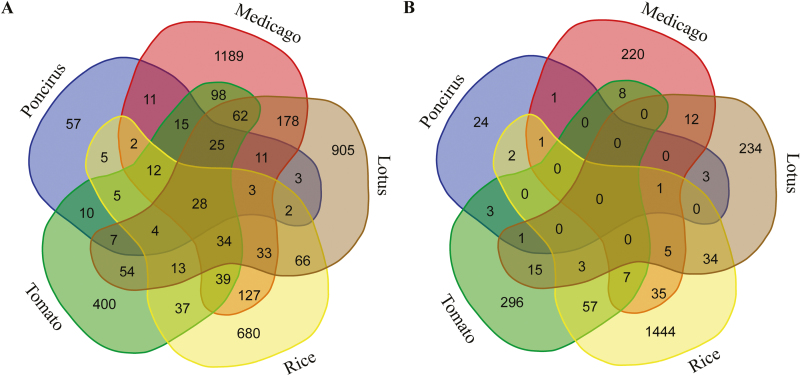
Distribution of up- and down-regulated orthogroups upon AMF colonization in *Poncirus trifoliata*, *Medicago truncatula*, *Lotus japonicus*, *Oryza sativa*, and *Solanum lycopersicum*. (A) Venn diagram showing the number of up-regulated orthogroups among the five AM host plants. (B) Venn diagram showing the number of down-regulated orthogroups among the five AM host plants. The transcriptome data in (A, B) are based on *M. truncatula* (Hogekamp *et al.*, 2011; Gaude *et al.*, 2012; Garcia *et al.*, 2017), *L. japonicus* (Handa *et al.*, 2015), *S. lycopersicum* (Fiorilli *et al.*, 2009; Sugimura and Saito., 2017), and *O. sativa* (Gutjahr *et al.*, 2015).

We first analysed genes that exhibited AMF-induced up-regulation. As shown in the Venn diagram in Fig. 2A, we identified 28 orthogroups, representing 41 (*O. sativa*), 50 (*P. trifoliata*), 63 (*L. japonicus*), 63 (*S. lycopersicum*), and 89 (*M. truncatula*) genes that showed up-regulation in all the five AM host plants. There were 106 orthogroups that contained up-regulated genes from at least four of the five plant species, and 72 orthogroups that contain up-regulated genes in at least three other plant species in addition to *P. trifoliata*. In total, 143 orthogroups representing 186 up-regulated *P. trifoliata* genes (75.9 % of all up-regulated genes) contained orthologous genes from at least one other species.

We tentatively defined those AMF-induced *P. trifoliata* genes that were members of orthogroups containing genes induced by AMF from at least two other plant species as the core set of *P. trifoliata* candidate genes important for AMS. This core set contained 153 up-regulated genes, which included the 21 AMS-associated genes and orthologs of 83 (representing 59 *P. trifoliata* genes) of the 138 *M. truncatula* genes conserved in AM-host plants, and three down-regulated genes (Supplementary Table S12). From this set, orthologs of 50 genes showed up-regulation in all five AM host plants. In addition to the *P. trifoliata* core set, we found 181 orthogroups containing genes that were induced in at least three plant species but not in *P. trifoliata*, which included two of the 24 AM-associated genes (Supplementary Table S8) and 19 of the 138 genes conserved in AM-host plants (Supplementary Table S10).

Of the 282 DEGs in *P. trifoliata*, 43 genes (33 up- and 10 down-regulated) did not have clear orthologous genes in any of the other four species, and 41 genes (27 up- and 14 down-regulated) did not have orthologs that were transcriptionally regulated in any of the other four species (Supplementary Table S11). A total of 1189, 905, 680, and 400 orthogroups representing more than 41.3%, 25.3%, 7.4% and 25.9% of the total number of up-regulated genes contained genes that were uniquely induced in *M. truncatula*, *L. japonicus*, *O. sativa*, and *S. lycopersicum*, respectively (Fig. 2A), in addition to 221, 368, 543, 142 up-regulated genes that did not have clear orthologous genes in the other species.

Interestingly, among the 37 *P. trifoliata* genes that showed down-regulation upon AMF colonization, we only identified one orthogroup (containing one *P. trifoliata* gene, Cs8g07230) that contained orthologous genes in the other species that were also down-regulated. There were relatively few orthologous genes that were commonly down-regulated between any three of these five species during AMS (Fig. 2B).

### Functional analysis of core genes

**Fig. 3.**
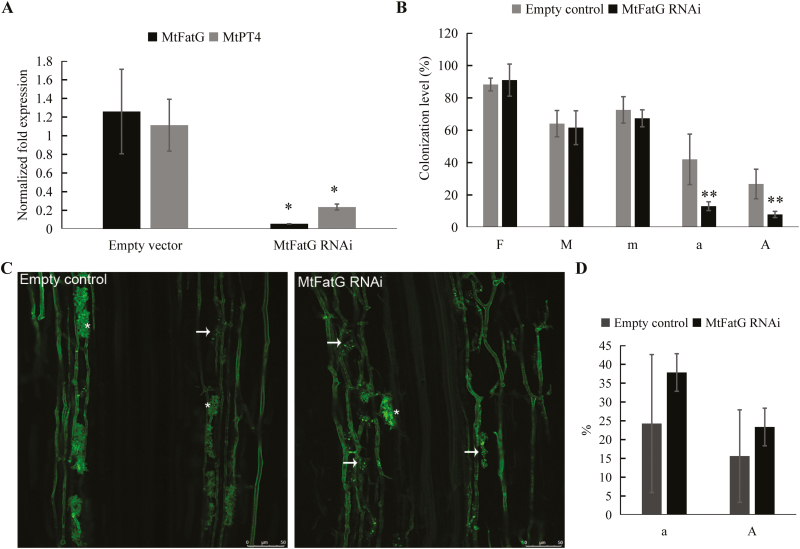
Silencing of *MtFatG* in *Medicago truncatula* impairs the development of arbuscules. (A) Transcription levels of *MtFatG* and *MtPT4* in *M. truncatula* RNAi transgenic roots and empty vector transgenic roots (control) based on qPCR analysis. Data are means (±SD) from five independent control transgenic roots and three independent *MtFatG*-RNAi transgenic roots. *MtEF* was used as the reference gene. Significant differences compared to the control were determined using Student’s *t*-test (*P<0.05). (B) Quantification of mycorrhization in *MtFatG*-RNAi and control roots, based on the method of Trouvelot *et al.* (1986). Data are means (±SD) from five independent control transgenic roots and six independent *MtFatG*-RNAi transgenic roots. F, frequency of mycorrhization in more than 200 root fragments of 1 cm each; M, intensity of infection in the total root system; m, intensity of infection in all mycorrhized root fragments; a, arbuscule abundance in mycorrhized root parts; A, arbuscule abundance in the total root system. Significant differences compared with the control were determined using Student’s *t*-test (**P<0.01). (C) Hairy roots of *M. truncatula* transgenic for an RNAi construct targeting *MtFatG* and empty vector controls were inoculated with *R. irregularis* and root segments were stained with WGA-Alexa fluor 488 followed by fluorescence imaging. Asterisks indicate well-developed arbuscules; arrows indicate degrading arbuscules. Scale bars are 50 μm. (D) Quantification of the abundance of degrading arbusulces in *MtFatG*-RNAi and control roots. Data are means (±SD) from five independent control transgenic roots and six independent *MtFatG*-RNAi transgenic roots. a, abundance of degrading arbuscules in all mycorrhized root parts; A, abundance of degrading arbuscules in the total root system. The data are representative results from two independent replicates.

Our *P. trifoliata* comparative transcriptomics approach revealed a core set of genes that transcriptionally responded to different AMF in several diverse plant species, which strongly suggested that they play key roles in the symbiosis. To test this, we focused on one of the core set of AMF-induced and arbuscule-enriched *P. trifoliata* genes, *FatG*, for functional analysis. Its putative ortholog in *M. truncatula* (*MtFatG*; Medtr4g097510) was highly induced in arbuscule-containing cells, as revealed by RNA-seq analyses of laser-microdissected mycorrhizal root tissue (Supplementary Fig. S4B, C; Gaude *et al.*, 2012; Zeng *et al.*, 2018). To determine the role of *FatG* in AMS, we generated transgenic *M. truncatula* roots expressing an RNAi construct targeting *MtFatG* and confirmed that they exhibited reduced expression to less than 5% of that detected in control roots, based on qRT-PCR analysis (Fig. 3A). Reduction of *MtFatG* did not significantly affect colonization levels by *R. irregularis* (Fig. 3B). However, the abundance of the mature/well-developed arbuscules was strongly reduced (indicated by asterisks in Fig. 3C); 13.03% (a%) in *MtFatG* RNAi roots compared to 41.97% in empty vector control roots (Fig. 3B). Correspondingly, *MtFatG*-RNAi roots tended to contain more collapsed arbuscules associated with septa (indicated by arrows in Fig. 3C) compared to control roots (Fig. 3D) (a%, *P*<0.2313; A%, *P*<0.3040; ANOVA). Lower levels of functional arbuscules in the RNAi roots were also indicated by lower expression levels of the arbuscule-specific marker *MtPT4* compared to control roots (Fig. 3A). These results demonstrated that *FatG* is required for the maintenance of arbuscule development during mycorrhizal symbiosis in *M. truncatula* and probably also in other plant species. This identifies a novel component of the mycorrhiza-induced regulon involved in fatty acid biosynthesis, which has been implicated in providing lipids as the carbon source for the fatty-acid auxotrophic AM fungi (Bravo *et al.*, 2017; Jiang *et al.*, 2017; Keymer *et al.*, 2017; Luginbuehl *et al.*, 2017).

## Discussion

In this study we present the first genome-wide examination of the transcriptional response of *Poncirus trifoliata*, the most common citrus rootstock, upon colonization by the AMF *Glomus versiforme*. Through comparative transcriptome analysis we identified a core set of AM-induced genes for AMS that are conserved between *P. trifoliata* and four model plant species. This corroborates the notion of an ancient symbiotic pathway that triggers a conserved expression program in evolutionarily diverse plants, in line with other studies that have compared AM-induced genes between plant species (Breuillin *et al.*, 2010; Tromas *et al.*, 2012).

Several factors affect/complicate the comparison of expression profiles between different transcriptome studies. These include, for example, differences in growth conditions, transcriptome profiling methods, and associated analysis parameters (microarray/Genechip versus RNA-seq, whole-root versus specific cell types isolated by laser-capture microdissection), as well as variable colonization levels and fungal identity. With respect to the latter, it has been shown that more than 50% of the AM-induced genes in *M. truncatula* roots differ depending on the identity of the fungal species, i.e. *G. mosseae* versus *R. irregularis* (Hohnjec *et al.*, 2005; Hogekamp *et al.*, 2011). Thus, part of the differences in expression profiles between the studies we have compared may be due to the use of different AMF species: *G. versiforme* in *P. trifoliata*, *R. irregularis* in *L. japonicus* (Handa *et al.*, 2015) and *O. sativa* (Gutjahr *et al.*, 2015), *R. irregularis* and *G. mosseae* in *M. truncatula* (Hogekamp *et al.*, 2011; Gaude *et al.*, 2012; Garcia *et al.*, 2017) and *S. lycopersicum* (Fiorilli *et al.*, 2009; Sugimura and Saito., 2017). This may in part contribute to the identification of 181 orthogroups that contained AMF-induced genes in at least three of the herbaceous plants but which lacked a *P. trifoliata*-induced gene. However, the fact that a conserved transcriptional program is used in different plants interacting with different AMF strengthens the notion that such differentially expressed genes probably play key general roles in the symbiosis.

In addition, the developmental status of the root system has been shown to impact the AM-dependent transcriptome. A recent comparison in rice showed a striking difference in the number of DEGs upon AMS depending on the root type (Gutjahr *et al.*, 2015). Rice produces three root types that differ in AM colonization levels: low-colonized crown roots, highly colonized large lateral roots, and non-colonized fine laterals. The large lateral roots show a strikingly lower number of DEGs compared to crown roots, even though the latter are less well colonized. In this respect it is worth noting that we only used non-woody lateral roots of *P. trifoliata* for the transcriptome analyses, in order to avoid older woody/lignifying roots for which it is difficult to extract good-quality RNA and which are not colonized by AMF. This may in part explain the relatively small number of DEGs that we found in *P. trifoliata* compared to some of the more extensively studied herbaceous plants.

Despite these issues, we hypothesize that a considerable number of the DEGs that were only detected in one plant species (7.4–41.3% of the total DEGs; Fig. 2A) reflect the true differential impact of AMS on the distinct physiological and developmental programs of the different host species. AMF-dependent transcriptional repression in particular seemed to be considerably different in different AM host plants, as only a few orthologous genes were found to be commonly down-regulated between three of the five species studied.

We noted that three of 24 functionally characterized AM-induced genes did not show up-regulation in the *P. trifoliata* transcriptome data. These were *MIG1*, *DIP1*, and *RFCb*. It has been reported that the GRAS-domain transcription factor MtMIG1 functions in both arbuscule development and cortical cell expansion (Heck *et al.*, 2016). Based on our phylogenetic analysis, we noted that while *M. truncatula* had five genes assigned to the MIG1 orthogroup, there are only two *P. trifoliata* genes (Cs9g02380 and orange1.1t01684) orthologous to *MtMIG1* (Supplementary Fig. S5). It is possible that strong constitutive expression of these two *P. trifoliata MIG1* genes is sufficient for arbuscule development during mycorrhization or that weak expression of these genes in arbuscules cells could not be detected using our whole-root samples. With regard to *DIP1* (also a GRAS-domain transcription factor; Yu *et al.*, 2014) and *RFCb* (a protein that has a domain shared with DNA replication factor; Bravo *et al.*, 2016), it is interesting to note that the *C. sinensis* genome lacked an ortholog of *DIP1*. In *L. japonicus* and *S. lycopersicum* the ortholog of RFCb was shown to be up-regulated, whereas this was not the case in rice.

Despite these differences, we detected a set of 153 *P. trifoliata* genes that were significantly induced upon mycorrhization and whose orthologs in at least two other plant species were similarly induced upon AMS. This set reflected a core genetic program that was induced independent of plant and fungal identity. We therefore consider this core set of genes as likely key candidates to play important roles in AM symbiosis, at least within angiosperms. Many (59) of the core genes that we identified have been shown by a previous phylogenomics approach to be strictly conserved in AM host plants but absent in non-host plants (Bravo *et al.*, 2016). In addition, our comparative transcriptome approach identified 94 conserved AMF-induced genes that were not identified by the phylogenomics-only approach (Bravo *et al.*, 2016). The reason for this is that in the latter study a more extensive set of plant species were compared and orthologous genes could be found in some non-host plants. The conserved transcriptional response that we found here suggests that such genes are also likely to play a role in AMS. This is supported by the functional characterization of several of these genes. For example, the H^+^-ATPase HA1 (represented by Cs5g08370) is required to energize nutrient uptake and arbuscule development in M. *truncatula* and *O. sativa* (Krajinski *et al.*, 2014; Wang *et al.*, 2014); the ABC transporter PDR1 (represented by orange1.1t01531) is required for transport of strigolactones and regulates the development of AM symbiosis in *Petunia hybrida* (Kretzschmar *et al.*, 2012); the N-acetlyglucosamine transporter NOPE (represented by orange1.1t00603) was shown to be essential for the initiation of AM symbiosis in rice and *Zea mays* (Nadal *et al.*, 2017); and the recently identified transcription factor MYB1 (represented by Cs6g08490) as well as its downstream cysteine proteases CP3, CP4/CP5 (represented by Cs2g15480, Cs2g15490, and Cs2g15700) and chitinases (represented by Cs8g17580, Cs9g14710, and orange1.1t00435) are associated with arbuscule degeneration in M. *truncatula* (Floss *et al.*, 2017). The core set of genes also includes the ketoacyl-ACP synthase LjDISI/MtKasII (represented by Cs5g01990), which is an essential component of the mycorrhiza-induced regulon involved in fatty acid biosynthesis (Jiang *et al.*, 2017; Keymer *et al.*, 2017). This regulon has been shown to include the AM-host conserved fatty acyl–acyl carrier protein (ACP) thioesterase FatM and the glycerol-3-phosphate acyltransferase RAM2, involved in 16:0 β-monoacylglycerol synthesis (Wang *et al.*, 2012; Bravo *et al.*, 2017; Jiang *et al.*, 2017; Luginbuehl *et al.*, 2017). Mutants in these genes all display defects in arbuscule development. We identified eight additional genes in the core set that are related to lipid metabolism (Supplementary Table S12). One of these, *FatG*, is a 3-ketocyl-ACP reductase that mediates reduction of the 3-keto group from acetoacetyl-(acp) to 2-oxohexadecanoyl-(acp), which is the next step following KASI/KASII in fatty acid synthesis (Kallberg *et al.*, 2002). Our observation that silencing of *FatG* in *M. truncatula* roots resulted in impaired arbuscule development suggests a conserved role for *FatG* in the fatty acid synthesis regulon to provide lipids to the fungus.

Overall, our results highlight the power of comparative transcriptomics to identify additional host genes and programs essential for AMS. The conserved AM-up-regulated genes presented here represent a valuable data set to guide future functional studies.

## Supplementary data

Supplementary data are available at *JXB* online.

Fig. S1. Mycorrhizal colonization levels and AM fungal structures in *P. trifoliata* roots.

Fig. S2. Assays for promoter activity of the *P. trifoliata Chit2*, *PMI2*, *Lipase3*, and *Exo70I* genes in transgenic *M. truncatula* hairy roots.

Fig. S3. Promoter activity of *P. trifoliata FatG* in the transgenic *M. truncatula* hairy roots.

Fig. S4. Gene expression of *PtrFatG* and *MtFatG* in mycorrhizal and non-mycorrhizal roots.

Fig. S5. A clade of the phylogenetic tree of MIG1 proteins.

Table S1. Summary of results of mapping to the reference genome.

Table S2. Gene-specific primers used in quantitative RT-PCR.

Table S3. List of construct and gene primers used in this study.

Table S4. Transcripts detected in all the six samples by RNA-seq.

Table S5. Differentially expressed genes in response to *G. versiforme* colonization in *P. trifoliata*.

Table S6. Gene expression levels detected by quantitative RT-PCR.

Table S7. List of 24 genes known to be functional in AMS.

Table S8. List of three genes known to be functional in AMS that were not AM-responsive in *P. trifoliata*.

Table S9. Distribution of the MYCS/CTTC motif in the promoters of five genes from *P. trifoliata* and *M. truncatula*.

Table S10. List of 19 AMS-conserved genes induced in at least three of the model species but not in *P. trifoliata*.

Table S11. AM-responsive genes only detected in *P. trifoliata* or with orthologous genes also induced in one of the four model species.

Table S12. List of a core set *P. trifoliata* genes showing similar AMF-dependent differential expression in at least two other plant species.

Supplementary Figure S1-S5

Supplementary Tables S1-S12

## Abbreviations:

AM: arbuscular mycorrhiza
AMF: arbuscular mycorrhizal fungi
AMS: arbuscular mycorrhizal symbiosis
DEG: the differentially expressed gene
