# Supplementary material for: Comparative transcriptome analysis of Poncirus trifoliata identifies a core set of genes involved in arbuscular mycorrhizal symbiosis
Source: J Exp Bot. 2018 Jul 26;69(21):5255–64. doi: 10.1093/jxb/ery283 (PMC6184448; doi:10.1093/jxb/ery283)
Supplement: Supplementary Figure S1-S5 [file ery283_suppl_supplementary_figure-s1-s5.pdf]

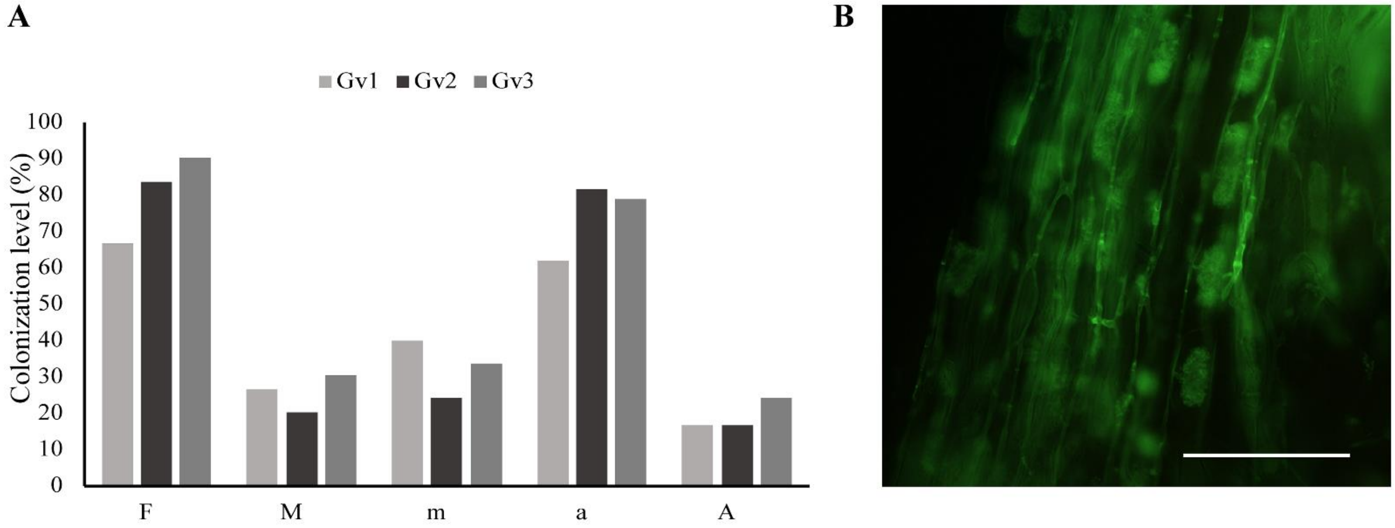


Fig. S1. Mycorrhizal colonization level and AM fungal structures in *Poncirus trifoliata* roots.
(A) Mycorrhizal colonization level of the three *Poncirus trifoliata* root samples inoculated with *Glomus versiforme* after 3 months; (B) Visualization of AM fungal structures in *Poncirus trifoliata* roots inoculated with *G. versiforme*. The roots were stained with WGA-alexa fluor 488 to visualization of AM fungal structures (green). Scale bars, 100 µm.

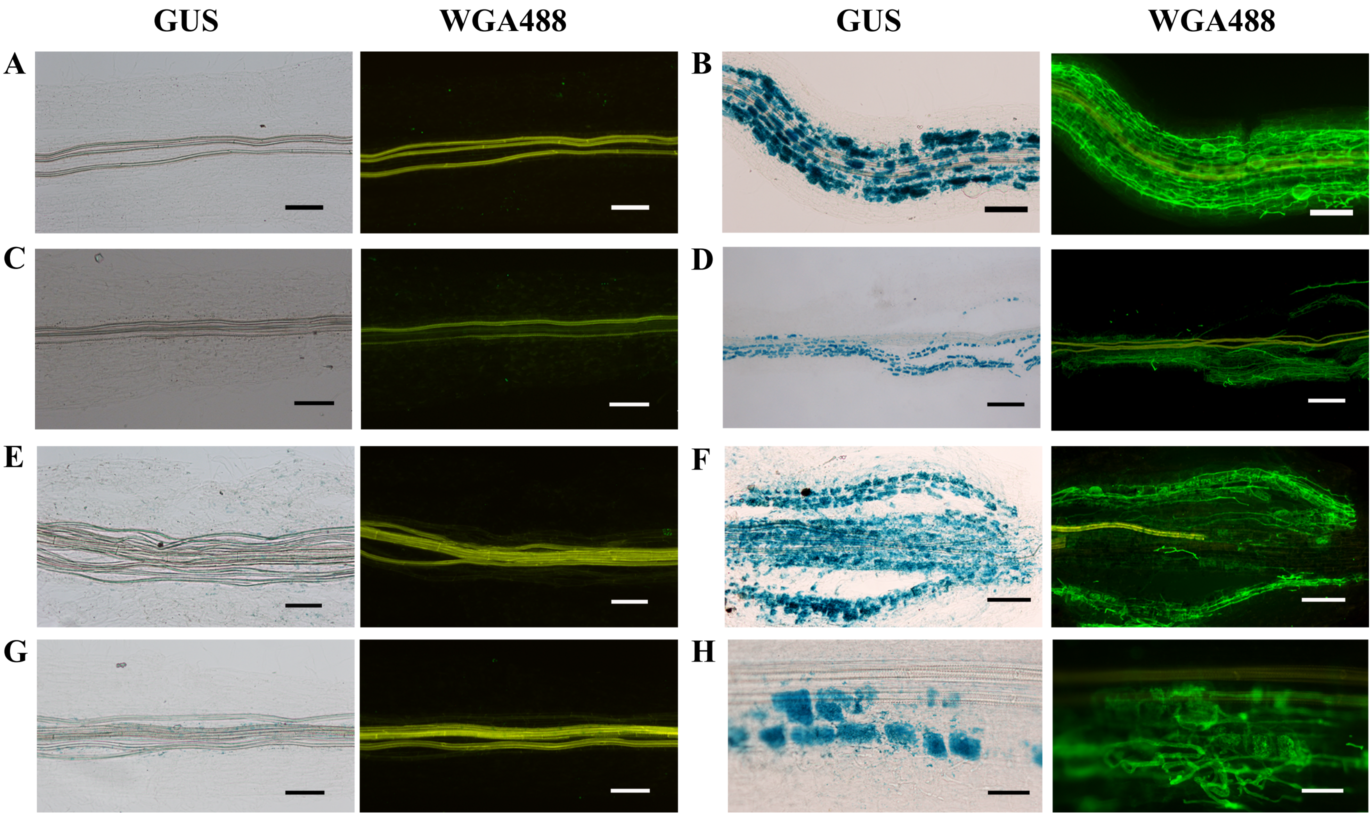


Fig. S2. Assays for promoter activity of the Poncirus trifoliata Chit2, PMI2, Lipase3 and Exo70I genes in transgenic Medicago truncatula hairy roots.
Medicago root segments expressing the gusAint gene under control of the promoters of Poncirus Chit2 (A, B), PMI2 (C, D), Lipase3 (E, F) and Exo70I (G, H) were assayed for GUS activity (left panel) and visualization of AM fungal structures with WGA Alexa Fluor 488 (right panel). (A), (C), (E) and (G) Transgenic hairy roots free from AMF. (B), (D), (F) and (H) Transgenic hairy roots colonized by AMF. Scale bars, 200 μm for (C) and (D); 150 μm for (B), 100 μm for (A), (F) and (G), 50 μm for (E) and (H).

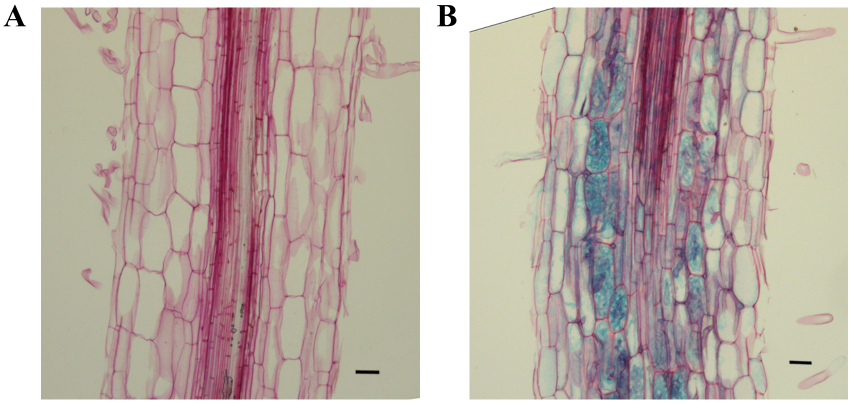


Fig. S3. Promoter activity of Poncirus trifoliata FatG in the transgenic Medicago truncatula hairy roots. (A) The root section of Medicago hairy roots transformed with PtrFatGpro::GUS were not inoculated by R.irregularis. Bars= 25 μm. (B) The root section of Medicago hairy roots transformed with PtrFatGpro::GUS were colonized by R.irregularis. Bars= 25 μm.

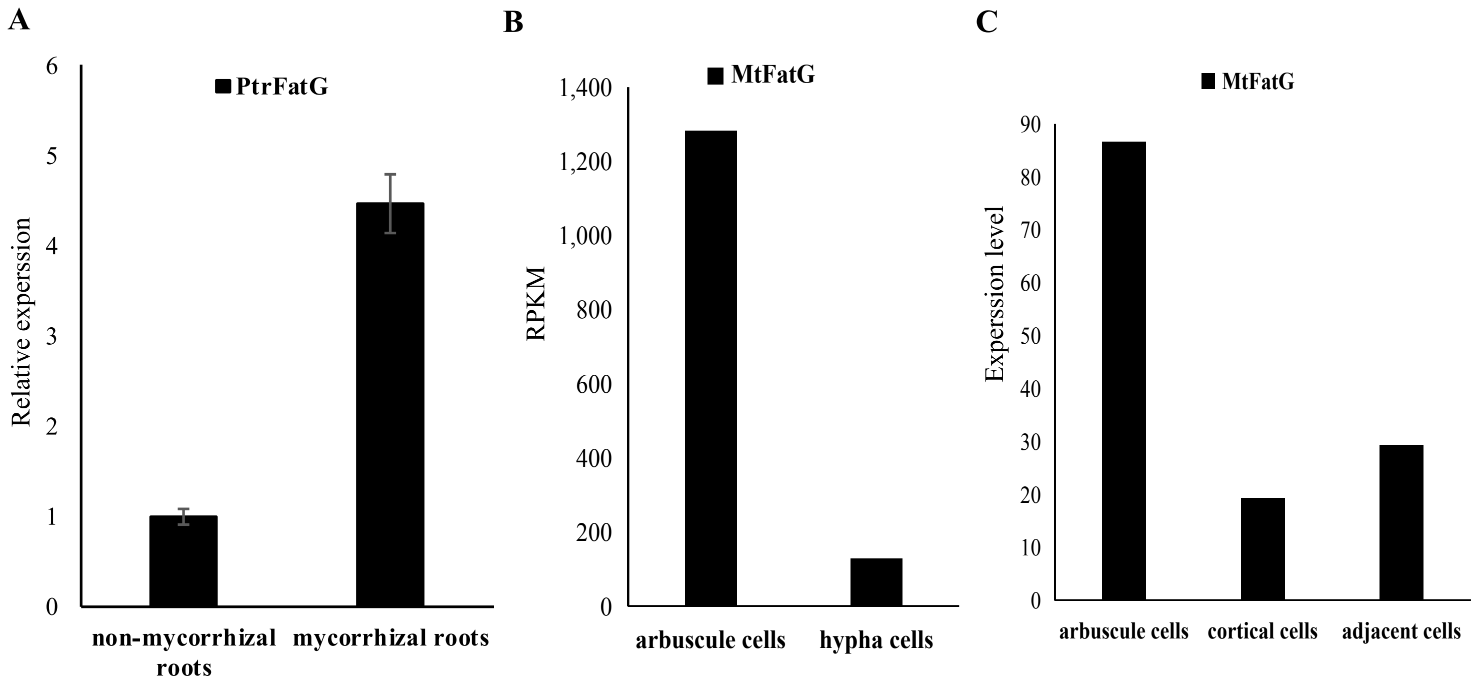


Fig. S4. Gene expression of *PtrFatG* and *MtFatG* in mycorrhizal roots and non-mycorrhizal roots. (A) qRT-PCR measurement of the relative expression level of *PtrFatG* in Poncirus trifoliata non-mycorrhizal and mycorrhizal roots. (B) Expression level of *MtFatG* in arbuscule cells and cortical cells containing hypha from mycorrhizal roots (Zeng et al., 2018). (C) The expression level of *MtFatG* in the different symbiotic cells dectected by laser capture micodisection data (Gaude et al., 2012).

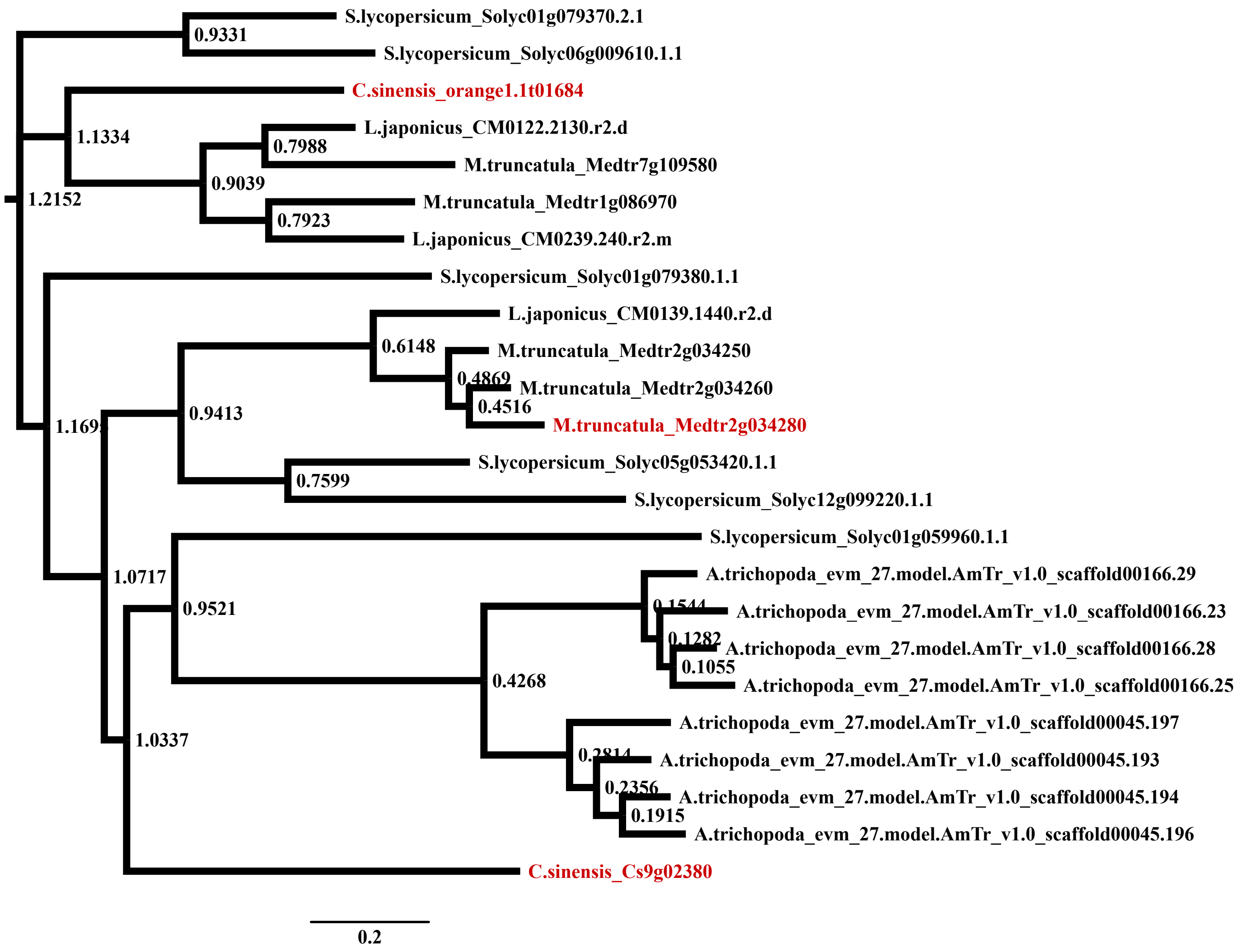


Fig. S5 A clade of the phylogenetic tree of MIG1 proteins.
This clade of phylogenetic tree is obtained from OthoFinder analysis, based on the genome of Medicago truncatula, Lotus japonicus, Solanum lycopersicum, Oryza sativa, Arabidopsis thaliana and the basal angiosperms Amborella trichocarpa. Medtr2g034280 is the gene ID of MIG.
